# Associations of Intensive Lifestyle Intervention in Type 2 Diabetes With Health Care Use, Spending, and Disability: An Ancillary Study of the Look AHEAD Study

**DOI:** 10.1001/jamanetworkopen.2020.25488

**Published:** 2020-11-24

**Authors:** Peter J. Huckfeldt, Chris Frenier, Nicholas M. Pajewski, Mark Espeland, Anne Peters, Ramon Casanova, Xavier Pi-Sunyer, Lawrence Cheskin, Dana P. Goldman

**Affiliations:** 1 Division of Health Policy and Management, University of Minnesota School of Public Health, Minneapolis; 2 Department of Biostatistics and Data Science, Wake Forest School of Medicine, Winston-Salem, North Carolina; 3 Keck School of Medicine of the University of Southern California, Los Angeles; 4 Columbia University, New York, New York; 5 George Mason University, Fairfax, Virginia; 6 Leonard D. Schaeffer Center for Health Policy and Economics, University of Southern California, Los Angeles; 7 School of Pharmacy, University of Southern California, Los Angeles; 8 Price School of Public Policy, University of Southern California, Los Angeles

## Abstract

**Importance:**

Intensive lifestyle interventions focused on diet and exercise can reduce weight and improve diabetes management. However, the long-term effects on health care use and spending are unclear, especially for public payers.

**Objective:**

To estimate the association of effective intensive lifestyle intervention for weight loss with long-term health care use and Medicare spending.

**Design, Setting, and Participants:**

This ancillary study used data from the Look AHEAD randomized clinical trial, which randomized participants with type 2 diabetes to an intensive lifestyle intervention or control group (ie, diabetes support and education), provided ongoing intervention from 2001 to 2012, and demonstrated improved diabetes management and reduced health care costs during the intervention. This study compared Medicare data between study arms from 2012 to 2015 to determine whether the intervention was associated with persistent reductions in health care spending.

**Exposure:**

Starting in 2001, Look AHEAD’s intervention group participated in sessions with lifestyle counselors, dieticians, exercise specialists, and behavioral therapists with the goal of reducing weight 7% in the first year. Sessions occurred weekly in the first 6 months of the intervention and decreased over the intervention period. The controls participated in periodic group education sessions that occurred 3 times per year in the first year and decreased to 1 time per year later in the trial.

**Main Outcomes and Measures:**

Outcomes included total Medicare spending, Part D prescription drug costs, Part A and Part B Medicare spending, hospital admissions, emergency department visits, and disability-related Medicare eligibility.

**Results:**

This study matched Medicare administrative records for 2796 Look AHEAD study participants (54% of 5145 participants initially randomized and 86% of 3246 participants consenting to linkages). Linked intervention and control participants were of a similar age (mean [SD] age, 59.6 [5.4] years vs 59.6 [5.5] years at randomization) and sex (818 [58.1%] women vs 822 [59.3%] women). There was no statistically significant difference in total Medicare spending between groups (difference, −$133 [95% CI, −$1946 to $1681]; *P* = .89). In the intervention group, compared with the control group, there was statistically significantly higher Part B spending (difference, $513 [95% CI, $70 to $955]; *P* = .02) but lower prescription drug costs (difference, −$803 [95% CI, −$1522 to −$83]; *P* = .03).

**Conclusions and Relevance:**

This ancillary study of a randomized clinical trial found that reductions in health care use and spending associated with an intensive lifestyle intervention for type 2 diabetes diminished as participants aged. Intensive lifestyle interventions may need to be sustained to reduce long-term health care spending.

**Trial Registration:**

ClinicalTrials.gov Identifier: NCT03952728

## Key Points

**Question:**

Is an intensive lifestyle intervention for type 2 diabetes associated with long-term health care use and Medicare spending?

**Findings:**

This ancillary study of a randomized clinical trial linked 2796 participants with type 2 diabetes in a randomized intensive lifestyle intervention with Medicare data. Among linked participants, the intervention was associated with reduced weight, improved diabetes control, and reduced health care costs during the intervention, but there was no reduction in total health care spending after the intervention.

**Meaning:**

These findings suggest that intensive lifestyle interventions targeted to patients with type 2 diabetes may need to be sustained to reduce long-term health care spending.

## Teaser

This ancillary study of a randomized clinical trial examines the long-term associations of an intensive lifestyle intervention for type 2 diabetes with health care use, spending, and disability.

## Introduction

About 9% of the US population has received a diabetes diagnosis, and treatment costs exceed $327 billion per year.^1^ Most of this diabetes is type 2, and primary and secondary prevention are cornerstones of the public health strategy.^2^ In particular, excess body weight is linked with worse glycemic control and elevated cardiovascular risk.^3^ As a result, there is significant interest in intensive lifestyle management with a focus on diet, physical activity, and weight loss to reduce the incidence and harm of type 2 diabetes.^4^ There are reasons to be optimistic. Individualized guidance on diet and exercise are effective at preventing diabetes in the first place and improving diabetes control and reducing cardiovascular risk factors for those with type 2 diabetes.^5,6,7,8^

Policy makers, insurers, and employers have taken notice. Versions of intensive lifestyle interventions (ILIs) have been incorporated into Medicare,^9^ workplace wellness programs,^10^ and patient-centered medical homes.^11^ However, these interventions are labor intensive and expensive, often involving lifestyle counselors, exercise specialists, dieticians, and administrative staff.^12,13^ Thus, it is important to demonstrate long-term efficacy and value, although some advocates assume it.^14^ Many studies find that interventions can reduce short-term health care use, but long-term estimates on health care use and costs often rely on simulations that make assumptions about long-term persistence of these short-term effects.^15,16^

In this study, we linked administrative data with a clinical trial to examine whether reductions in health care spending were sustained after an ILI ended. The data were obtained from the Look AHEAD (Action for Health in Diabetes) study, one of the largest ILIs performed to date. Beginning in 2001, 5145 patients aged 45 to 76 years with type 2 diabetes who were overweight or obese were randomized to ILI or to a control group that received diabetes support and education.^8^ Although the intervention did not significantly reduce its primary outcome, a composite of death from cardiovascular causes, nonfatal myocardial infarction or stroke, or hospitalization for angina, the intervention successfully reduced participants’ weight, increased physical fitness and functional status, lowered hemoglobin A_1c_ (HbA_1c_), and increased probability of diabetes remission, among other clinical benefits.^8,17,18,19,20^ However, the intervention was also expensive, costing $2865 per patient per year in the first year and gradually decreasing to $1120 per patient per year in years 5 to 9 (in 2012 dollars), whereas the diabetes support and education received by the control group cost less than $202 per patient year in the first year and $103 in years 5 to 9.^13^ The intervention also reduced hospitalizations, prescription drug use, and total health care costs during the intervention period by $5280 per patient (in 2012 dollars).^21^ An outstanding question is whether these reductions persisted after the intervention, particularly given that the intervention was not cost-saving during the trial.

## Methods

### Study Overview and Design

This ancillary study to the Look AHEAD randomized clinical trial examines the association of an ILI for weight loss targeted to patients with type 2 diabetes with long-term health care use, Medicare spending, and disability insurance enrollment. The Look AHEAD study ended the intervention in September 2012 but continued to follow participants. As a part of the observational studies following the intervention period, the Look AHEAD study obtained written informed consent to perform linkages with administrative records. We linked study participants to Medicare records (for those who consented to such a linkage) to estimate outcomes in the year the intervention ended (2012) and the 3 following years (2013 to 2015). The University of Southern California and Wake Forest institutional review boards approved the data linkage, and the University of Minnesota institutional review board deemed it exempt from review because University of Minnesota researchers did not work with direct identifiers. Our manuscript proposal to the Look AHEAD study, including our proposed outcome measures and analyses, is available in the Manuscript Proposal Supplement 1. The trial protocol for the Look AHEAD study is published elsewhere.^8^ This study is reported following Consolidated Standards of Reporting Trials (CONSORT) reporting guideline, as applicable to a secondary analysis of clinical trial data.

The Look AHEAD study randomized participants within 16 study sites to either the ILI group or a control group (diabetes support and education). Randomization occurred between 2001 and 2004. The most intensive portion of the intervention, and the greatest weight loss, occurred during the first year, when participants had weekly sessions with counselors, dieticians, exercise specialists, and behavioral health staff. By the fourth year, individual contacts occurred on a monthly basis, as well as group classes provided throughout the year.^8^ The control group received 3 educational sessions per year in the first 4 years, and annually thereafter.

### Data Linkage and Study Population

**Figure 1.**
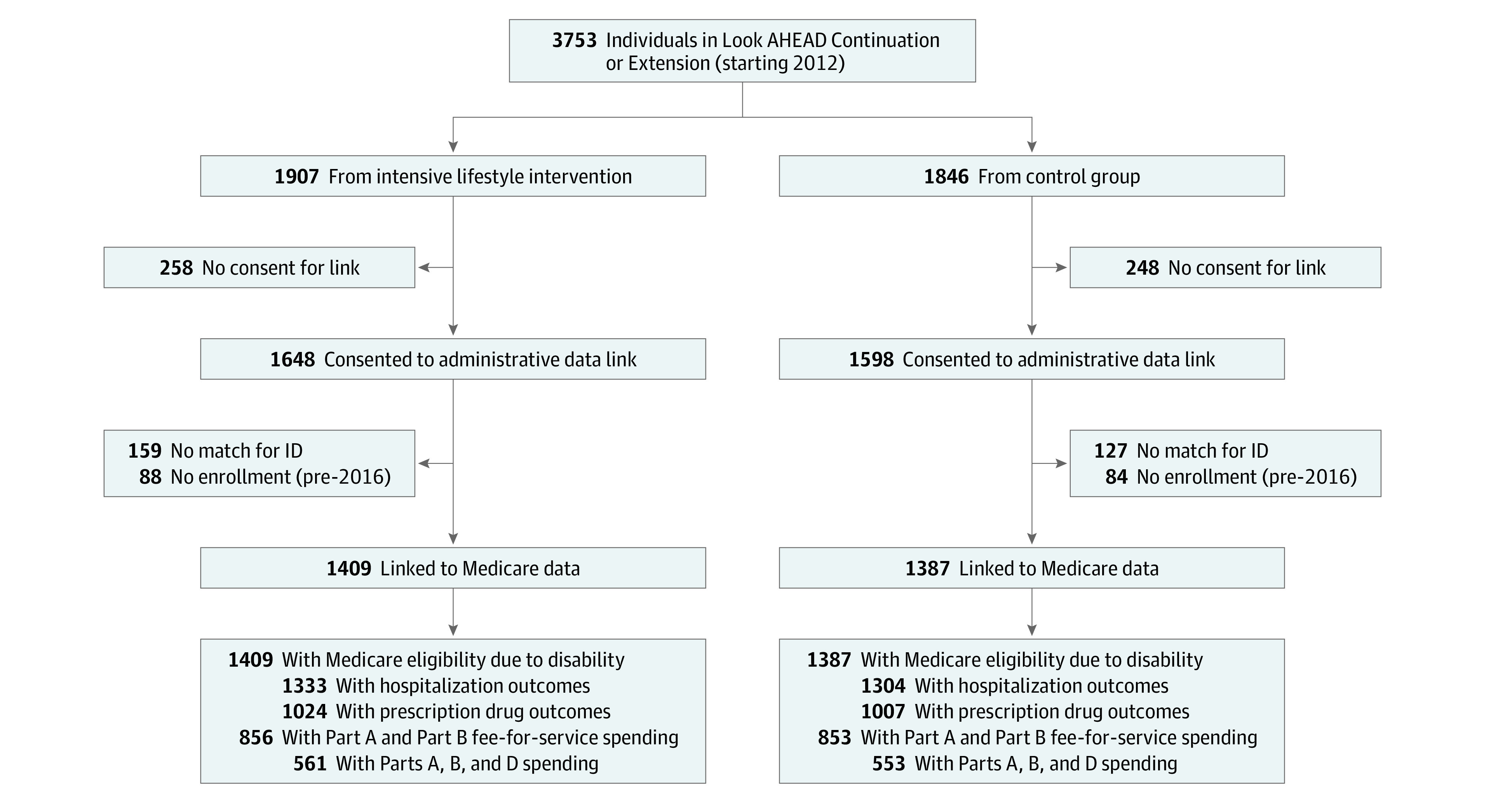
Flow Diagram of Look AHEAD Participants in Sample

Our analysis compared Medicare outcomes between the ILI and control groups after the intervention. Not all study participants were given the opportunity to consent to data linkages, as the queries were conducted as part of 2 cohort studies following the intervention (the Look AHEAD-Continuation study and the Look AHEAD-Extension study). Of the original 5145 Look AHEAD participants, 3753 (1907 intervention and 1846 controls) participated in the Look AHEAD Continuation or Extension studies (Figure 1). Of these, 3246 participants (1648 intervention participants and 1598 control participants) consented to administrative data linkages and 2796 participants (1409 intervention participants and 1387 control participants) were successfully linked to Medicare data (comprising 75% of participants in the Continuation and Extension studies and 86% of participants consenting to linkages). More details of the linkage are available in the eAppendix in Supplement 2.

### Data Sources

We used the Medicare Master Beneficiary Summary File to determine participants’ eligibility, Medicare Advantage enrollment, and spending by service category. One concern about analyses using Medicare data is the absence of claims data for participants in Medicare Advantage. We measured hospital admissions and emergency department (ED) use for enrollees in Medicare Advantage using patient-level data from the 2012 to 2014 Healthcare Effectiveness Data and Information Set. Medicare Advantage plans are required to report these data to the National Committee for Quality Assurance for nearly all Medicare Advantage enrollees; thus, these data capture health care use for most enrollees in our sample. We constructed identical measures of hospitalizations and ED visits for fee-for-service Medicare enrollees from Medicare claims, following prior research comparing fee-for-service Medicare and Medicare Advantage.^22^ We linked the Medicare data with Look AHEAD trial data, which includes baseline participant characteristics, intervention arm assignment, and clinical outcomes during the study.

### Study Measures

We examined the long-term associations of the ILI with measures of health care use, medical care spending, prescription drug spending, and Medicare eligibility owing to disability or end-stage renal disease. We preregistered our study outcomes on ClinicalTrials.gov.

The unit of observation for all health care use and spending measures was at the person-year level. Measures of health care use included whether participants had any hospital admission or ED visit and the number of hospitalizations and ED visits. Annual medical care spending measures included Medicare Part A (hospitals, postacute care, and other health care facilities) and Part B (professional payments, some home health care, medical equipment, laboratory tests, mental health services, and ambulance services). We measured annual prescription drug spending using total drug costs incurred in Medicare Part D. Specifically, this outcome measures total Part D drug costs for a given year, including the ingredient cost, dispensing fees and sales tax, but not manufacturer discounts or rebates.^23^ We also investigated annual beneficiary out-of-pocket spending on prescription drugs in Medicare Part D. Total annual Medicare spending was defined as the sum of Medicare Part A and Part B spending and total Part D prescription drug costs. Functional status was measured using the reason for initial Medicare eligibility, reflecting that individuals who are eligible for Social Security Disability Insurance become eligible for Medicare after 2 years.^24^ In addition, individuals with end-stage renal disease, which can be a complication of type 2 diabetes, may be eligible for Medicare prior to age 65 years. A small minority of participants were enrolled in Medicare at the beginning of the trial. Thus, to the extent that the ILI improved functional status or reduced diabetes-related kidney disease, the trial could have decreased subsequent Medicare eligibility owing to disability or end-stage renal disease and increased Medicare enrollment owing to age.

A key challenge in our analysis was that not all components of health care spending were available for all study participants, based on enrollment in traditional Medicare vs Medicare Advantage and enrollment in Part D drug coverage. Specifically, annual medical care spending measures (excluding prescription drugs) were only available for fee-for-service Medicare enrollees (ie, those enrolled in Medicare Parts A and B), and prescription drug costs were only available for enrollees in Medicare Part D prescription drug coverage. Consequently, total annual Medicare spending (ie, the sum of medical care spending and prescription drug costs) was only available for linked Look AHEAD participants enrolled in both fee-for-service Medicare and Medicare Part D.

As a result, in our primary analysis, we estimated the association of ILI with health care use and spending measures for individuals enrolled in fee-for-service Medicare (Parts A and B) and Part D prescription drug coverage. For this sample, we were able to observe total Medicare spending, as well as each component of Medicare spending and health care use. Notably, the probability of inclusion in the primary analysis sample was nearly identical between ILI and control group person-years (1662 person-years [31.0%] vs 1592 person-years [30.3%]; *P* for difference = .43) and included 3254 person-years between 2012 and 2015 (for spending measures) and 2327 person-years between 2012 and 2014 (for health care use measures). Thus, while this sample comprised a minority of linked participants, inclusion in this group appeared unrelated to intervention status and the sample offered sufficient statistical power to detect meaningful effects.

Next, we investigated whether the effects of ILI for the primary analysis sample were representative of the broader linked sample by estimating analyses using the largest possible sample for each component of health care use and Medicare spending. For hospital admissions and ED visits, this analysis included 3705 linked person-years (95%) in the control group and 3792 linked person-years (96%) in the ILI group (*P* for difference = .46) (eTable 1 in Supplement 2). For prescription drug costs, the sample included all participants enrolled in Medicare Part D, comprising 3463 linked person-years (66%) in the control group and 3594 linked person-years (67%) in the ILI group (*P* = .22). Finally, for Part A and Part B spending, our sample included 2824 person-years (54%) in the control group and 2874 person-years (54%) in the ILI group (*P* = .89).

### Statistical Analysis

First, we examined whether our linked sample (ie, those who consented and for whom we were able to successfully obtain Medicare records) was representative of the broader Look AHEAD study. We compared the characteristics of the linked sample (2796 participants) with originally randomized participants (5145 participants) and then assessed whether the linked sample replicates the effects of ILI on weight and HbA_1c_ documented for the original sample.^8^

Next, we estimated the associations of ILI with hospital and ED use and Medicare spending. We used generalized linear models for health care use and spending outcomes (eAppendix in Supplement 2).^25^ We used logit models to estimate Medicare disability status and other binary outcomes. For the person-year–level analysis of annual health care use and spending, we calculated cluster-robust SEs at the person level. We calculated heteroskedasticity-robust SEs for the person-level disability analysis. All analyses controlled for demographic characteristics (ie, age, sex, and self-reported race/ethnicity), clinical status at initial randomization (ie, obesity status, HbA_1c_, hypertension, and cardiovascular disease), socioeconomic status at initial randomization (ie, education and income), and initial study site (using fixed effects). We also controlled for months of Medicare coverage for those who may have enrolled mid-year and year-fixed effects in models investigating health care use and Medicare spending.

We also estimated year-specific associations of the intervention with selected measures of health care use and spending.

Data were analyzed using Stata statistical software version 15.0 (StataCorp). *P* values were 2-sided, and statistical significance was set at .05. Analysis began in December 2018 and was completed in September 2020.

## Results

We found no statistically significant differences in baseline characteristics between linked ILI and control group participants (Table 1). The 1409 ILI and 1387 control participants were of a similar age (mean [SD] age at randomization, 59.6 [5.4] years vs 59.6 [5.5] years) and sex (818 [58.1%] women vs 822 [59.3%] women). The linked sample was also similar to full originally randomized Look AHEAD sample along most characteristics (eTable 2 in Supplement 2). Two clinic sites in Arizona and New Mexico declined participation in the data linkages; as a result, the proportion of American Indians were lower in our analysis (exact data not displayed to comply with the CMS Cell Size Suppression Policy).

**Table 1.** Baseline Characteristics of Linked Participants

Characteristic	No. (%)	
	Control (n = 1387)	Intervention (n = 1409)
Age, mean (SD) y	59.6 (5.5)	59.6 (5.4)
Women	822 (59.3)	818 (58.1)
Race/ethnicity		
White, non-Hispanic	915 (66.0)	938 (66.6)
Black, non-Hispanic	234 (16.9)	227 (16.1)
Hispanic	195 (14.1)	188 (13.3)
Weight, mean (SD), kg	100.3 (18.1)	100.1 (19.1)
BMI, mean (SD)	35.8 (5.7)	35.6 (5.9)
Obesity status		
Reference range to overweight	200 (14.4)	225 (16.0)
Obese	896 (64.6)	901 (63.9)
Very obese	291 (21.0)	283 (20.1)
Hemoglobin A_1c_, mean (SD), % of total hemoglobin	7.2 (1.1)	7.2 (1.1)
Duration of diabetes, mean (SD) y	6.6 (5.9)	6.8 (6.8)
Hypertension	1169 (84.3)	1173 (83.3)
History of cardiovascular disease	169 (12.2)	178 (12.6)
Employed	916 (66.0)	891 (63.2)
Education		
≤High school	304 (21.9)	297 (21.1)
Some postsecondary	510 (36.8)	479 (34.0)
≥College graduate	573 (41.3)	633 (44.9)

Abbreviation: BMI, body mass index (calculated as weight in kilograms divided by height in meters squared).

SI conversion factor: To convert hemoglobin A_1c_ to proportion of total hemoglobin, multiply by 0.01.

### Associations of ILI With Weight and HbA_1c_ During and After the Trial

**Figure 2.**
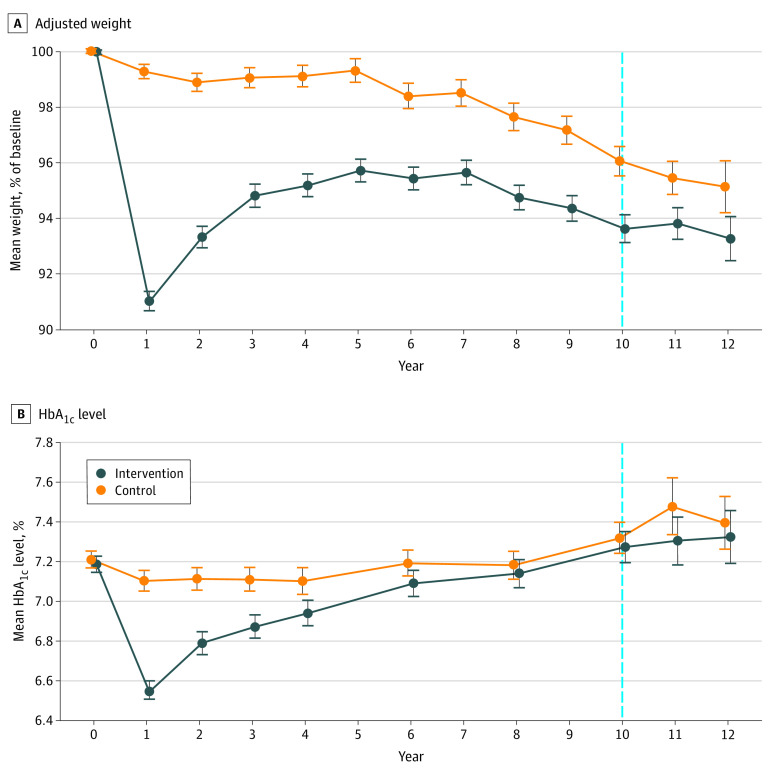
Adjusted Weight and Hemoglobin A_1c_ (HbA_1c_) During the Trial Stratified by Intervention Status Each point represents the mean weight or HbA_1c_ in each year relative to randomization separately for intervention and control participants, adjusted for baseline participant characteristics and study site. Error bars indicate 95% CIs; vertical line, indicates median intervention length (10 years).

We found similar associations of ILI with weight and HbA_1c_ for the linked sample compared with the full Look AHEAD study during and after the trial (Figure 2).^26^ During our sample period from 2012 to 2015, the linked ILI group continued to have lower weight than the control group (adjusted difference: −1.9% [95% CI, −2.6% to −1.2%] of initial body weight; *P* < .001). However, there was no significant difference in HbA_1c_ between 2012 and 2015. These results are discussed in more detail in the eAppendix in Supplement 2.

### Associations of ILI With Health Care Use and Spending

**Figure 3.**
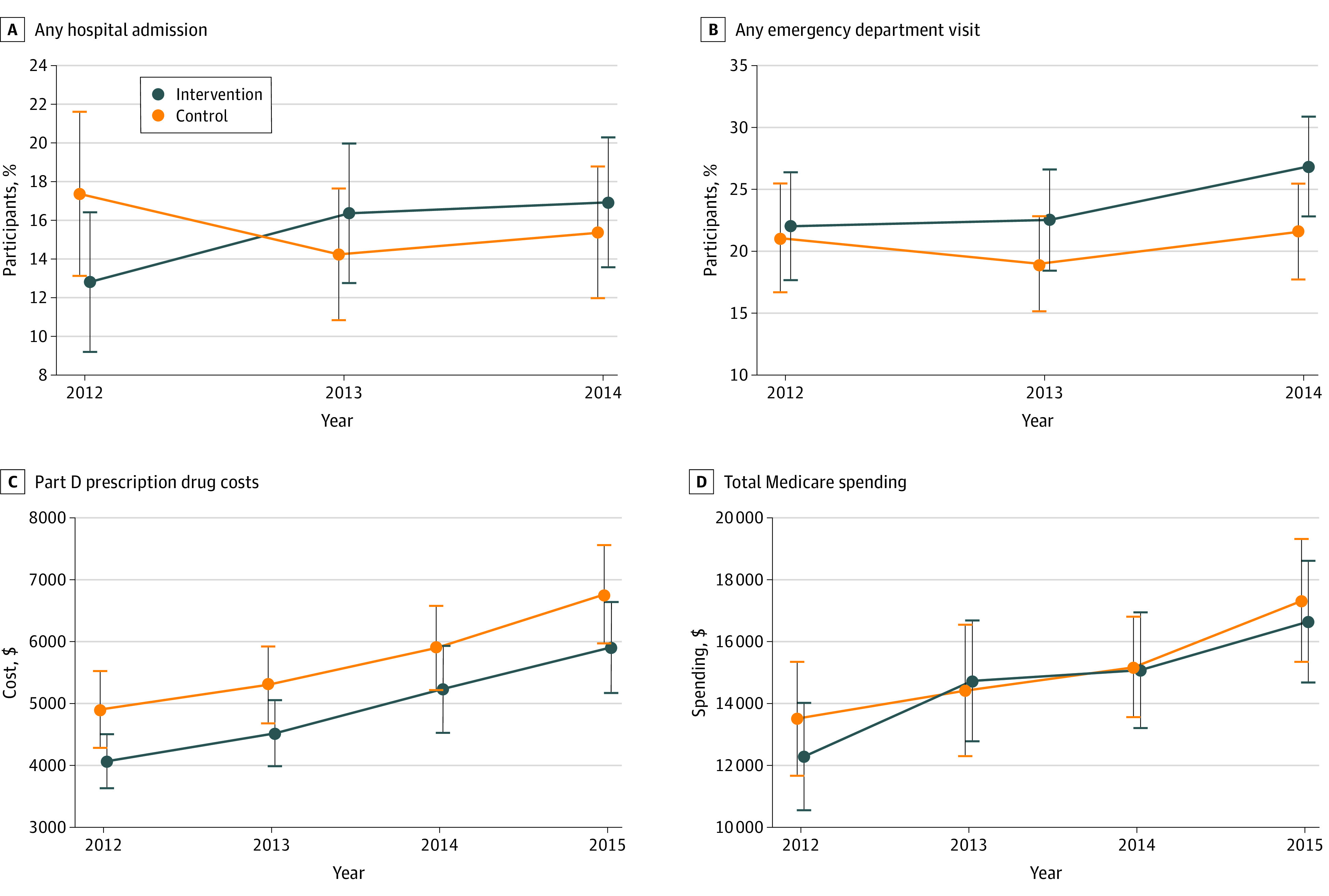
Adjusted Health Care Use and Spending by Intervention Status Each point represents the utilization outcome in each year separately for intervention and control participants, adjusting for baseline participant characteristics and study site. Spending measures presented in 2015 dollars. Error bars indicate 95% CIs.

For our primary analysis sample (ie, fee-for-service Medicare enrollees with Part D prescription drug coverage), a similar percentage of person-years in the ILI and control groups had any hospitalization (188 person-years [15.8%] vs 174 person-years [15.3%]; adjusted difference: 0.1 [95% CI, −3.2 to 3.3] percentage points; *P* = .96) and any ED visit (285 person-years [23.9%] vs 234 person-years [20.6%]; adjusted difference: 3.5 [95% CI, −0.3 to 7.2] percentage points; *P* = .07) (Table 2). Similarly, there was no difference in the annual number of hospital admissions or the annual number of ED visits. We found no significant difference in Part A spending (adjusted difference: $488 [95% CI, −$715 to $1691]; *P* = .43) but found higher Part B spending for the ILI group (adjusted difference: $513 [95% CI, $70 to $955]; *P* = .02). We found that ILI participants had lower mean (SD) Part D prescription drug costs compared with the control group ($5046 [$8199] vs $5849 [$9122]; adjusted difference: −$803 [95% CI, −$1522 to −$83]; *P* = .03) and lower mean (SD) Part D out-of-pocket spending ($1353 [$2197] vs $1536 [$1891]; adjusted difference: −$196 [95% CI, −$383 to −$8]; *P* = .04). However, there was no difference in mean (SD) total Medicare spending ($15 005 [$23 522] vs $15 096 [$21 821]; adjusted difference: −$133 [95% CI, −$1946 to $1681]; *P* = .89). We also estimated year-specific ILI vs control group differences in the probability of hospital admission and ED visits, Part D prescription drug costs, and total Medicare spending. We displayed each outcome by year and intervention status, adjusting for baseline characteristics and study site (Figure 3). None of the year-specific adjusted outcomes differed significantly by intervention status. The only statistically significant year-specific differences were for Part D costs in 2012 (adjusted difference for ILI versus control group: −$836.6 [95% CI, −$1517.7 to −$155.6]; *P* = .02) and 2013 (adjusted difference: −$785.6 [95% CI, −$1529.9 to −$41.3]; *P* = .04),which became insignificant in subsequent years.

**Table 2.** Health Care Use and Spending by Group^a^

Outcome	Group		Adjusted difference (95% CI)	Difference, % (95% CI)^b^	*P* value
	Control	Intervention			
Hospital and emergency department use (2012-2014)					
Any hospital admission, No. (%)	174 (15.3)	188 (15.8)	0.1 (−3.2 to 3.3)	0.5 (−20.6 to 21.7)	.96
Hospital admissions per participant-year, mean (SD)	0.22 (0.65)	0.22 (0.63)	−0.01 (−0.07 to 0.05)	−3.9 (−30.2 to 22.4)	.77
Any ED visit, No. %	234 (20.6)	285 (23.9)	3.5 (−0.3 to 7.2)	16.9 (−1.2 to 35.0)	.07
ED visits per participant-year, mean (SD)	0.29 (0.75)	0.32 (0.66)	0.03 (−0.04 to 0.09)	9.2 (−13.5 to 31.8)	.43
Medicare spending (2012-2015), mean (SD) $^c^					
FFS spending					
Part A	5529 (16 152)	5687 (15 183)	488 (−715 to 1691)	8.8 (−12.9 to 30.6)	.43
Part B	3717 (4593)	4273 (7824)	513 (70 to 955)	13.8 (1.9 to 25.7)	.02
Total gross costs for Part D prescription drugs	5849 (9122)	5046 (8199)	−803 (−1522 to −83)	−13.7 (−26.0 to −1.4)	.03
Total beneficiary out-of-pocket payments for Part D prescription drugs	1536 (1891)	1353 (2197)	−196 (−383 to −8)	−12.7 (−24.9 to −0.5)	.04
Total Medicare spending	15 096 (21 821)	15 005 (23 522)	−133 (−1946 to 1681)	−0.9 (−12.9 to 11.1)	.89

Abbreviation: FFS, fee-for-service.

^a^ Outcomes include annual measures of health care use and spending. Means are calculated across person-years. The estimation sample for spending measures included 3254 person-years from 2012 to 2015 with observed FFS Medicare enrollment (Parts A and B) and Part D prescription drug coverage. The estimation sample for hospital and emergency department use included 2327 person-years from 2012 to 2014 with observed FFS Medicare enrollment (Parts A and B) and Part D prescription drug coverage.

^b^ Reports adjusted difference and confidence interval divided by the control mean.

^c^ Spending results expressed in constant 2015 dollars.

In ancillary analyses, we estimated differences in each component of health care use and spending between the ILI and control groups using the largest samples possible for each measure (eTable 3 in Supplement 2). Similar to the primary analysis sample, we found no difference in hospital admissions, ED visits, and Part A spending; we found lower Part D prescription drug costs (adjusted difference: −$538 [95% CI, −$993 to −$83]; *P* = .02) and Part D beneficiary out-of-pocket drug spending (adjusted difference: −$171 [95% CI, −$282 to −$60]; *P* = .002). In contrast to the primary sample, we found no significant difference in Part B spending. Finally, there was no difference in the proportion of ILI and control group participants who were originally eligible for Medicare because of end-stage renal disease or Social Security Disability Insurance enrollment (150 participants [10.7%] vs 142 participants [10.3%]; adjusted difference: 0.3 [95% CI, −1.7 to 2.4] percentage points; *P* = .77).

## Discussion

In an era of increasing health care costs associated with chronic illness, it is imperative to identify interventions to reduce long-term spending without harming patient care. To this end, this ancillary study linked data from a large randomized clinical trial to Medicare claims to identify the long-term associations of intensive lifestyle management for patients with type 2 diabetes. Similar to the overall study, we found significant reductions in weight and HbA_1c_ in the linked intervention group compared with the linked control group, with reductions in weight persisting beyond the intervention period. Despite this, we found no postintervention differences in hospital admissions or ED use, Medicare Part A spending, or eligibility for Medicare owing to enrollment in Social Security Disability Insurance or end-stage renal disease. While we found that the intervention participants had lower gross costs for Part D drugs from 2012 to 2015, they also had higher Part B spending, and there was no difference in total Medicare spending.

Our study is not the first to investigate whether improving diabetes management can reduce health care spending. Observational studies have found that reductions in body mass index and HbA_1c_ were correlated with reductions in health care spending among patients with type 2 diabetes.^27,28^ Other studies have found that interventions successfully managing diabetes were associated with reduced short-term health care spending.^15,16^ However, we found that the benefits of successful diabetes interventions may not persist after interventions end. Notably, our ability to track health care use and spending was facilitated by the linkage of trial participants with Medicare data and highlights the utility of administrative data sources for tracking the long-term associations of interventions with patient outcomes.

Our results are also related to recent work using randomized clinical trials to estimate the associations of workplace wellness programs with health care use and spending.^29,30^ These programs include components that are similar to the intensive lifestyle intervention we studied, with registered dieticians and other practitioners providing counseling on nutrition and physical activity. In contrast, the wellness programs were targeted broadly at all employees, and both studies found small and statistically insignificant associations of the programs with health outcomes, health care use, and spending. Our results, paired with earlier estimates from the Look AHEAD trial, show that targeting lifestyle interventions to at-risk populations (ie, patients with type 2 diabetes) may be important for successfully improving chronic disease management and reducing health care use and spending, but spending reductions may not persist beyond the intervention period.^21^

While our analysis found that reductions in prescription drug spending persisted beyond the trial period, the differences became statistically insignificant over time. In addition, the benefits in terms of reduced hospitalizations during the intervention did not persist beyond the intervention period, although hospitalizations were measured during the trial using validated self-reports and medical record reviews rather than Medicare data.^21^ This result may imply that the benefits of lifestyle intervention diminish over time, particularly as the patient cohort ages and faces other health risks. Extending interventions may be a potential approach to achieve persistent effects of ILI; however, more evidence is needed to determine whether this is an effective strategy to reduce long-term health care use and spending. Alternatively, there is evidence that the Look AHEAD ILI was associated with reduced bone mineral density and increased the risk of frailty fractures.^31^ More work is needed to investigate whether such outcomes may have increased hospitalizations, potentially offsetting the other clinical benefits of Look AHEAD.

Given that microvascular and macrovascular complications of diabetes continue to develop 15 to 25 years after diagnosis,^32^ the full benefits of the ILI in terms of reduced health care use and spending may have yet to be observed. In addition, starting in 2015, clinical guidelines began favoring new, more expensive drugs as second-line treatments for type 2 diabetes, including sodium-glucose cotransporter-2 inhibitors and glucagon-like peptide 1 agonists.^33^ To the extent that the ILI and control groups had varying needs for these new agents, this may also have affected differences in prescription drug costs and total health care spending after our sample period. Finally, although the costs of the ILI may not have been offset by other health care savings during and after the intervention, the considerable clinical and functional benefits of ILI during and after the intervention period may have been worth the costs. Formal cost-effectiveness analysis is necessary to weigh the costs and benefits of the ILI.

### Limitations

Our study had several limitations. First, because the questions about data linkage manifested after the study was under way, we were only able to link 54% of the original cohort to Medicare records. To the extent that linked ILI participants differed from linked control participants along unobserved attributes that were correlated with long-term health care use, such imbalance could introduce bias our results. However, we were able to link a nearly identical percentage of ILI and control participants to Medicare data (54% in each group), the linked participants were similar to the original cohort along observed characteristics, the linked intervention and control groups were balanced across observed characteristics, and most importantly, the intervention had similar effects on body weight and HbA_1c_ for linked participants as the original cohort.

Second, we were not able to observe all outcomes for all linked participants. In particular, we only observed total Medicare spending among fee-for-service Medicare enrollees with Part D prescription drug coverage. However, we did not observe differential enrollment by study arm, suggesting that this did not bias our estimates.

Third, we were only able to observe health care use recorded in the Medicare program. One of the Look AHEAD trials’ clinic sites included a Veterans Health Administration facility. Individuals with both Veterans and Medicare coverage may have received care at Veterans Health Administration facilities that did not appear in Medicare claims. Only approximately 5% of the sample came from this site, and our results were unchanged removing this site from the sample.

Fourth, the Look AHEAD trial recruited volunteers with type 2 diabetes who could complete a fitness test and were motivated to participate in the trial.^8^ Thus, the outcomes associated with ILI could differ in a broader population with type 2 diabetes.

## Conclusions

This ancillary study found that a randomized clinical trial of an ILI was not associated with reduced total Medicare spending in the years immediately following the intervention. These results suggest that ILIs may need to be sustained for reductions in health care costs to persist. However, some of the benefits of the trial may have yet to be observed, implying the importance of continued evaluation.

## Notes

Supplement 1. **Manuscript Proposal**

Supplement 2. **eTable 1.** Sample Sizes by Analytic Sample **eTable 2.** Baseline Characteristics of Full Sample and Linked Participants **eTable 3.** Health Care Use and Spending by Intervention Group, 2012 to 2015 **eAppendix.** Supplementary Methods and Results

Supplement 3. **Data Sharing Statement**
