# Supplementary material for: Associations of Intensive Lifestyle Intervention in Type 2 Diabetes With Health Care Use, Spending, and Disability: An Ancillary Study of the Look AHEAD Study
Source: JAMA Netw Open. 2020 Nov 24;3(11):e2025488. doi: 10.1001/jamanetworkopen.2020.25488 (PMC7686866; doi:10.1001/jamanetworkopen.2020.25488)
Supplement: Supplement 1. — Manuscript Proposal [file jamanetwopen-e2025488-s001.pdf]

Look AHEAD ANCILLARY STUDY Proposal Template

The following areas are to be addressed in the proposal. The proposal is limited to 5 pages or less (using 11 point font).
Submit your proposal to Carrie Williams (CCWillia@WakeHealth.edu) and
the Chair of the Ancillary Studies Committee, John M. Jakicic, PhD (jjakicic@pitt.edu)

NOTE: The submission is to be received at least 3 months in advance of any planned grant submission deadline. Submissions received <3 months in advance are not guaranteed to be reviewed and a decision made prior to the grant submission deadline.

1. **Date:** August 13, 2014 *(revised August 28, 2014)*

2. **Title of Ancillary Study:** The Long Term Economic Benefits of Health Improvement: Evidence from an RCT of an Intensive Lifestyle Intervention

3. **Principal Investigator:** Dana Goldman, Ph.D.
Institution: University of Southern California
Address: USC Schaeffer Center for Health Policy and Economics
University Gateway 100C
Los Angeles, CA 90089
E-mail Address: dpgoldma@healthpolicy.usc.edu
Phone Number: (213) 821-7948

Co-Principal Investigator: Peter Huckfeldt, Ph.D.
Institution: University of Minnesota School of Public Health
Address: Division of Health Policy & Management
420 Delaware St. S.E., MMC 729
Minneapolis, MN 55455
E-mail Address: huckfeld@umn.edu
Phone Number: (916) 203-9409

4. **Look AHEAD PI who is a Co-Investigator on this Project:**
Anne Peters, USC LookAHEAD site

5. **Additional Co-Investigators and E-mail Addresses:**

6. **Science Background, Rationale, Significance**:

The long-term health consequences of obesity, poor glycemic control, and cardiovascular risk factors among patients with type 2 diabetes are increasingly understood. In contrast, the economic impacts of poor diabetes control in terms of long-term health care costs, disability, and labor market productivity remain unclear. In particular, there has been a long-running debate about whether health determines economic outcomes, or vice versa (1, 2). Existing studies often use observational data, where unobserved factors may influence both disease control and economic outcomes.

The Look AHEAD study (Action in Health for Diabetes) is the largest randomized intensive lifestyle intervention for weight loss for type 2 diabetes ever conducted. Look AHEAD randomized two study arms: an Intensive Lifestyle Intervention (ILI) versus periodic education (Diabetes Support and Education (DSE)) across 5,145 patients with type 2 diabetes in 16 sites across the United States, with the goal of investigating the effects of intensive lifestyle intervention and weight loss on cardiovascular morbidity and mortality (3). Look AHEAD has demonstrated the efficacy of an ILI in achieving weight loss; in year 1, the ILI group lost 8.5% of initial weight relative to a 0.6% reduction for the DSE group. While some of the weight was regained in later years, at year 8 ILI participants remained 4.7% below their initial weight relative to a 2.1% reduction in the DSE group, with the difference being statistically significant (4). The ILI group has exhibited other health improvements, including reductions in cardiovascular risk factors (e.g., HbA1c, blood pressure, and triglycerides) (5); reduction in sleep apnea (6); increased health related quality of life (7); and higher rates of remission of type 2 diabetes (8), among others (9).

Such improvements in cardiovascular risk factors and health outcomes may have lead to improved economic outcomes among participants.

We propose research linking Medicare claims and Social Security Administration administrative records to Look AHEAD participants. This linkage will allow for a fuller accounting of the economic benefits of intensive lifestyle interventions, in terms of delayed retirement, longer-term health care costs and adverse health outcomes, and application and receipt of Social Security Disability Insurance. In addition, the random assignment of the ILI, and associated weight loss and improvement and cardiovascular risk, provides a unique and valuable opportunity to better understand the influence of health on economic outcomes for patients with type 2 diabetes. Finally, by comparing the occurrence of health events in Medicare data and Look AHEAD clinical records, we can evaluate the reliability of Medicare claims data as a tool for post-clinical trial follow-up[1].

7. **Main Hypothesis, Aims:**

The goal of the proposed ancillary study is to advance knowledge on the economic impacts of obesity, type 2 diabetes, and poor cardiovascular health. The broad hypothesis of the study is that the reductions in weight and improvements in health status from the ILI, described above, will lead to improved economic outcomes. Our specific aims are as follows:

Aim 1. Complete a linkage of Look AHEAD study participants with Medicare claims and Social Security Administration administrative records.

Aim 2. Estimate the impact of the Intensive Lifestyle Intervention (ILI) on medium-term Medicare spending and economic outcomes.

Aim 3. Model the long-term economic effects of the ILI in terms of value to society, based on estimates in Aim 2.

Aim 4. Evaluate the utility of administrative data as a means of post-clinical trial follow-up.

8. **Methods:**
   a. **Existing data you anticipate requesting**

We will request Social Security and Medicare record numbers for Look AHEAD participants who have consented to provide this information for linkage with Medicare claims and Social Security databases. For our analysis, we will request LookAHEAD study data including: (1) baseline demographic and health information on the Look AHEAD participants, (2) weight over the course of the study, (3) physical fitness when available, (3) laboratory data (e.g., HbA1c, HDL, LDL, triglycerides, and blood pressure), (4) health events over the course of the study (e.g., AMI, stroke), and (5) any information on health care costs over the course of the study.

   b. **If requesting biological samples (stored blood), provide details on the number of samples, time periods requested, amount of sample to be used, and details on how the samples will be used.**

We will not be requesting biological samples.

   c. **New data you anticipate collecting**

We anticipate collecting three new sources of administrative data: (1) Medicare Fee For Service claims data, (2) Social Security Administration disability insurance data, and (3) Social Security Administration employment data.

[1] NIDDK has expressed particular interest in exploring this question.

The study team has extensive experience handling sensitive administrative data, including linking clinical trial participants with Medicare data (10).

PHI data will be handled in a manner that is consistent with the policies of the Clinical Centers and informed consent processes. This will involve the data being retained by the Coordinating Center and to be handled in a manner that conforms to the final Data Use Agreements from CMS and SSA. Deviations from this plan will require approval by Look AHEAD prior to implementation.

Medicare FFS claims data

The study will submit an application to the CMS Privacy Board to obtain Medicare data for consenting patients. The research team has extensive experience with this process. In addition, Dr. Huckfeldt will work directly with ResDAC in the University of Minnesota School of Public Health, the contractor managing CMS Privacy Board submissions, to facilitate this application. Upon receiving CMS Privacy Board and IRB approval, we will obtain Fee For Service claims records from Medicare Part A (inpatient) and B (outpatient) data, which will cover claims for hospitalizations, post acute care facilities, outpatient hospitals and ambulatory surgery centers, professional services, home health care, durable medical equipment, and hospice. We will obtain pharmacy claims for patients from Medicare Part D. We will also obtain Medicare enrollment and coverage information for consenting patients. While claims will be available only for the ~75% of Medicare beneficiaries with Fee For Service (i.e. Part A and B) coverage, we will observe hospitalizations and Medicare enrollment information for Look AHEAD participants with coverage through Medicare Advantage (Part C).

Social Security Administration- Disability Insurance and Retirement Data

We will employ an approach similar to the linkage of the Health and Retirement Study with Social Security Administrative Records (11). In the preferred scenario, the study would securely transfer a list of consenting Social Security Numbers and study IDs to the Social Security Administration. The Social Security Administration would then extract and securely transfer the requested data with study IDs back to the Coordinating Center. This limited dataset (with SSNs removed) would be the base data for the analysis. An alternative approach would involve the analysis occurring at the Social Security Administration.

We will link the Look AHEAD study participants to the Social Security NUMIDENT file, the master file for all Social Security Number (SSN) applications. Using the validated SSN from NUMIDENT, we will link Look AHEAD participants to the Detailed Earnings Record (DER) from the Master Earnings File (MEF). The MEF contains annual earnings by detailed source, including self-employment income and deferred compensation, derived from IRS tax records. Additionally, we will link the Look AHEAD participants to the Master Beneficiary Record (MBR), which includes all beneficiary and payment information for the Social Security Disability Insurance (SSDI) program, and the Supplemental Security Record (SSR), which includes information on Supplemental Security Income (SSI) applications and recipients.

d. Sample size and power justification

Table 1 displays the results of power tests under different sample assumptions and outcomes. The minimum detectable difference will depend in part on the number of patients consenting to release their Medicare and social security numbers for the data linkage. The third column uses the number of consents as of July 1, 2014 (n=2,666). The fourth column uses the expected accrual of consents (n=4,366). In each case, we include minimum detectable difference for one-sided tests. The null hypotheses are that spending, hospital admissions, and SSDI applications in the ILI group will be greater than or equal to the DSE group, and that employment in the ILI group will be less than or equal to the DSE group.

The first row displays results for total annual health care spending. We will be able to measure total annual Medicare spending for participants with Medicare Part A and B coverage. Thus, we first reduce the total sample size by 20% (reflecting that 80% of Look AHEAD Participants were aged 65 or older as of 7/31/13) and then by 25% (reflecting that 25% of beneficiaries are covered by Medicare Advantage Part C), leading to an approximate sample size of 800 in each arm under current accrual and 1307 in each arm under expected accrual. This may understate the number of Medicare beneficiaries among LookAHEAD participants, depending on participation in the Social Security Disability Insurance program (which grants Medicare eligibility to individuals under age 65) and because additional participants will obtain Medicare eligibility over the course of the study. We use the mean and standard deviation of log-transformed health care spending for LookAHEAD participants separately calculated for the ILI and DSE group as an approximation for annual Medicare spending. Under current sample accrual, we would be able to measure approximately a 9.3% reduction in health care spending in the ILI group relative to the DSE group. Under the expected sample accrual we would be able to detect a 7.3% reduction in health care spending.

The second row displays results for "any hospitalization in the past year", based on the proportion of respondents with hospitalizations in the 2010 Medicare Current Beneficiary Survey among those with type 2 diabetes and aged 65 to 88 years old. We will be able identify hospitalizations for all Medicare patients (including Part C) in Medicare claims, thus we only reduced the sample size by 20%, reflecting the 20% of study participants who are not yet 65 years old. We will be able to detect a 3.0 to 3.9-percentage point reduction in hospital discharges, depending on sample accrual, with power equal to 0.80.

The third row examines the probability of applying for Social Security Disability Insurance (SSDI), based on the proportion of individuals with diabetes aged 50 or older reporting application in the 2013 National Health Interview Survey. We will examine SSDI application for all Look AHEAD participants. We estimate that we will be able to detect a 3.8 to 4.9-percentage point difference in SSDI application from a mean of 0.451.

Look AHEAD baseline data show that 62.8% of participants were employed at the start of the study. As shown in the fourth row of Table 1, we will be able to detect a 3.7 to 4.8-percentage point increase in employment.

Table 1. Estimated minimum detectable differences between ILI and DSE given available power sizes

Outcomes	Mean (Standard deviation)	Smallest difference detectable with current sample accrual, power=0.80 (n=2,666)	Smallest difference detectable with expected sample accrual, power=0.80 (n=4,366)
Log annual health spending [1]	ILI: 8.78 (0.76)<br>DSE: 8.86 (0.73)	9.3%	7.3%
Admitted to hosp (in past year) [2]	0.162	3.9 percentage points	3.0 percentage points
Applied for Social Security Disability Insurance (ever) [3]	0.451	4.9 percentage points	3.8 percentage points
Employed [4]	0.628	4.8 percentage points	3.7 percentage points

[1] Mean and standard deviation calculated from Look AHEAD.
[2] Proportion calculated using the 2010 MCBS.
[3] Calculated from 2013 National Health Interview Survey.
[4] Employed percentages from baseline LookAHEAD data.

9. **Burden (include plan to support burden and costs):**

The LookAHEAD re-consent form includes the following language: "I agree to provide my social security number and Medicare number so that the Look AHEAD Coordinating Center at Wake Forest University Health Sciences may search national databases for information about my health any time before 12/31/2020." Medicare and SSA disability records pertain directly to health, and employment information pertain indirectly to health, thus we expect to be able to complete the database linkages without obtaining additional consent from participants. The ability to complete linkages without re-consent greatly reduces the burden and cost of the study to participants and clinics. We continue to explore this issue with the relevant IRBs for the study and will only pursue the linkage of outcomes that do not require additional consent.

a. **Participants**
We anticipate no burden or cost on the part of patients.

b. **Clinic**
We anticipate no burden or cost on the part of clinics.

c. **Coordinating Center**
We will request study data and help facilitating the database linkage through the secure transmission of Social Security and Medicare numbers. The coordinating center will also retain the study databases, as described in (8c) above. We will work with the Coordinating Center during the proposal process to incorporate funding for all requested activities.

10. **Resources**
    a. **State clearly any resources you may need from the Coordinating Center (limited to data transfer from WFUHS)**
    We will not need any additional resources other than those listed under 9.
    b. **State clearly any identifiable needs from the clinics**
    We do not anticipate any needs from clinics.
11. **Safety:**
    a. **Risk to participants**
    The only risk to participants is the unintentional release of identifiable data.

    b. **Steps to mitigate or minimize**
    This risk will be minimized through the stringent data policies and security infrastructure in place at the Coordinating Center data facility. All analysis will be completed on limited or de-identified datasets on secure servers by approved project staff.

12. **Funding Mechanism:**
    a. **Granting agency:** NIDDK
    b. **Submission Deadline:** October 2014
    c. **RFA/PA is applicable:** We are open to submitting this proposal as an R01 or through another mechanism. We appreciate guidance on this question from the Ancillary Studies Committee.

13. **Cost for data analysis by Coordinating Center:**
    We will work with the Coordinating Center during the proposal process to determine a budget for data analysis.

References

1. Adams P, Hurd MD, McFadden D, Merril A, Ribeiro T. Healthy, wealthy, and wise? Tests for direct causal paths between health and socioeconomic status. Journal of Econometrics 2003;112(1):3-56.
2. Smith JP. Health Bodies and Thick Wallets: The Dual Relation Between Health and Economic Status. J Econ Perspect. 1999;13(2):144-68.
3. Look AHEAD Research Group. Look AHEAD (Action for Health in Diabetes): design and methods for a clinical trial of weight loss for the prevention of cardiovascular disease in type 2 diabetes. Control Clin Trials. 2003;24:610-28.
4. Look AHEAD Research Group. Eight-Year Weight Losses with an Intensive Lifestyle Intervention: The Look AHEAD Study. Obes Res. 2014;22(1):5-13.
5. Wing RR, Lang W, Wadden TA, Safford M, et al. Benefits of Modest Weight Loss in Improving Cardiovascular Risk Factors in Overweight and Obese Individuals with Type 2 Diabetes. Diabetes Care. 2011;34:1481-6.
6. Kuna ST, Reboussin DM, Borradaile KE, Sanders MH, et al. Long-Term Effect of Weight Loss on Obstructive Sleep Apnea Severity in Obese Patients with Type 2 Diabetes. Sleep. 2013;36(5):641-9. .
7. Williamson DA, Rejeski J, Lang W, Van Dorsten B, al. e. Impact of a weight management program on health-related qualtiy of life in overweight adults with type 2 diabetes. Arch Intern Med. 2009;169(2):163-71.
8. Gregg EW, Chen H, Wagenknecht L, et al. Association of an Intensive Lifestyle Intervention with Remission of Type 2 Diabetes. JAMA. 2012;308(23):2489-96.
9. Look AHEAD Research Group. Look AHEAD bibliography. Available from: https://lookaheadtrial.org/public/Bibliography.pdf.
10. Goldman DP, Berry SH, McCabe CH, et al. Incremental treatment costs in national cancer institute-sponsored clinical trials. JAMA. 2003;289(22):2970-7.
11. Olson JA. Linkages with Data from Social Security Administrative Records in the Health and Retirement Study. ORES Working Paper Series. 1999;Number 84.

Look AHEAD ANCILLARY STUDY Proposal Template

The following areas are to be addressed in the proposal. The proposal is limited to 5 pages or less (using 11 point font). Submit your proposal to Carrie Williams (CCWillia@WakeHealth.edu) and the Chair of the Ancillary Studies Committee, John M. Jakicic, PhD (jjakicic@pitt.edu)

NOTE: The submission is to be received at least 3 months in advance of any planned grant submission deadline. Submissions received <3 months in advance are not guaranteed to be reviewed and a decision made prior to the grant submission deadline.

14. **Date: December 3, 2018**

15. **Title:** The long-term effects of intensive lifestyle intervention on Medicare eligibility, hospital admissions, and prescription drug use

16. **Name of proposer and full contact information:** (and Look AHEAD collaborator for proposers outside of the Look AHEAD network)

Peter Huckfeldt, huckfeld@umn.edu, (612) 301-1320

Dana Goldman, dpgoldma@healthpolicy.usc.edu, 213-863-4492

17. **Proposed writing group members:** (Note this is a starting point; members may be added or subtracted with the final writing group being approved by the P&P Committee.)

Peter Huckfeldt (UMN), Dana Goldman (USC), Nick Pajewski (Wake Forest), Mark Espeland (Wake Forest), Anne Peters (USC), Chris Frenier (UMN)

18. **Background/Rationale**:

Look AHEAD tested whether participants assigned to an intensive lifestyle intervention (ILI) for weight loss in type 2 diabetes exhibited reductions in cardiovascular morbidity and mortality, relative to a control group receiving usual care and diabetes support and education.[1,2] During the 12-year intervention period, the ILI led to persistent reductions in weight, waist circumference, and hemoglobin A1c and improvements in physical fitness.[3] In addition, the ILI led to reductions in hospitalizations, hospital days, and prescription drug spending during the intervention period (through 2012).[4] However, the long-term effects on health care use and spending remain unknown.

In an ongoing ancillary study, we are investigating the effects of the ILI on economic outcomes, during and after the study. As a part of this study, we linked ~2,900 consenting Look AHEAD participants to Medicare databases. In the proposed manuscript, we will investigate the long-term effects of ILI on emergency department visits, hospitalizations, and pharmaceutical spending using Medicare data from 2012-2015, the three-year period immediately following the intervention period. We are focusing on these categories of health care use because: (1) they are key drivers of overall health care spending, (2) the ILI led to reductions in costs in these three categories during the intervention period,[4] and (3) they are consistently available in Medicare data for both beneficiaries with traditional fee-for-service coverage and private Medicare Advantage enrollees during our sample period.

By 2012 nearly all Look AHEAD participants were eligible for Medicare due to age (over 65). To the extent that Look AHEAD participants developed severe disabilities, they may have gained Medicare eligibility earlier through enrollment in Social Security Disability Insurance (SSDI). Thus, we will also test for differences in the Medicare enrollment channel between ILI and DSE participants, specifically whether the groups enrolled through SSDI at different rates.

19. **Main Hypothesis/Study Questions:**

- Did the ILI lead to long-term changes in emergency room visits, hospitalizations, and prescription drug use?
- Were ILI participants less likely to become eligible for Medicare due to disability?

20. **Study design and analysis (address each point separately):**

a. Description of the study design

This study will compare measures of health care use and Medicare enrollment between ILI and DSE participants after the conclusion of the ILI.

b. Inclusion/exclusion criteria

This study will include ~2,900 Look AHEAD participants who consented to administrative data linkages and were successfully matched to Medicare databases.

c. Outcome and other variables of interest with specific reference to the time of their collection

For Look AHEAD participants enrolled in fee-for-service Medicare, we are able to observe nearly all categories of health care use. However, among Look AHEAD participants enrolled in private Medicare Advantage plans, we are able to observe a more limited set of measures of health care use. Approximately 25% of Look AHEAD participants were enrolled in private Medicare Advantage plans at some point during our sample period. Our analysis will focus primarily on measures of health care use that are calculable for Medicare beneficiaries with traditional fee-for-service coverage and Medicare Advantage enrollees. The study outcomes, measure units, and sources of data for Medicare Advantage and fee-for-service enrollees are displayed in Table 1 below.

First, we will identify whether study enrollees became eligible for Medicare through age (i.e. turning 65) versus through disability insurance and/or End Stage Renal Disease, in order to test whether the ILI reduced the probability that Look AHEAD participants have sufficiently severe disabilities to qualify for SSDI. This information is included in the Medicare Beneficiary Summary File for all Medicare enrollees, regardless of coverage type. We will construct measures of hospital and emergency department use using Medicare inpatient and outpatient claims data for fee-for-service enrollees and annual patient-level HEDIS measures for Medicare Advantage enrollees. Finally, measures of annual prescription drug use are available for all fee-for-service and Medicare Advantage enrollees enrolled in standalone Part D plans or Medicare Advantage Part D plans in the Medicare Beneficiary Summary File Cost and Utilization supplement.

We will also conduct secondary analyses describing total Medicare spending for ILI and DSE participants enrolled in fee-for-service Medicare over the entire sample period (2001-2015) and compare these data to Look AHEAD self-reported spending when available.

Look AHEAD ANCILLARY STUDY Proposal Template

The following areas are to be addressed in the proposal. The proposal is limited to 5 pages or less (using 11 point font).
Submit your proposal to Carrie Williams (CCWillia@WakeHealth.edu) and
the Chair of the Ancillary Studies Committee, John M. Jakicic, PhD (jjakicic@pitt.edu)

NOTE: The submission is to be received at least 3 months in advance of any planned grant submission deadline. Submissions received <3 months in advance are not guaranteed to be reviewed and a decision made prior to the grant submission deadline.

Table 1. Main study outcomes

		Data sources	
	**Units**	**Medicare Advantage**	**Fee-for-service**
**1. Original Reason for Medicare Eligibility**			
Eligible through Old Age and Survivors Insurance	1 or 0 at person-level	Master Beneficiary Summary File- Base Segment, 2012-2015	Master Beneficiary Summary File- Base Segment, 2012-2015
Eligible through Disability Insurance Benefits	1 or 0 at person-level	Master Beneficiary Summary File- Base Segment, 2012-2015	Master Beneficiary Summary File- Base Segment, 2012-2015
Eligible through ESRD	1 or 0 at person-level	Master Beneficiary Summary File- Base Segment, 2012-2015	Master Beneficiary Summary File- Base Segment, 2012-2015
**2. Hospitalization outcomes**			
Any inpatient discharges	1 or 0 in each person-year	HEDIS patient-level files, 2012-2015	Inpatient Research Identifiable File, 2012-2015
Total inpatient discharges	Count per person-year	HEDIS patient-level files, 2012-2015	Inpatient Research Identifiable File, 2012-2015
Total inpatient days	Days per person-year	HEDIS patient-level files, 2012-2015	Inpatient Research Identifiable File, 2012-2015
Any medicine discharges	1 or 0 in each person-year	HEDIS patient-level files, 2012-2015	Inpatient Research Identifiable File, 2012-2015
Total medicine discharges	Count per person-year	HEDIS patient-level files 2012-2015	Inpatient Research Identifiable File 2012-2015
Total medicine days	Days per person-year	HEDIS patient-level files 2012-2015	Inpatient Research Identifiable File 2012-2015
Any surgical discharge	1 or 0 in each person-year	HEDIS patient-level files 2012-2015	Inpatient Research Identifiable File 2012-2015
Total surgical discharges	Count per person-year	HEDIS patient-level files 2012-2015	Inpatient Research Identifiable File 2012-2015

Total surgery days	Days per person-year	HEDIS patient-level files 2012-2015	Inpatient Research Identifiable File 2012-2015
Any ED visits	1 or 0 in each person-year	HEDIS patient-level files 2012-2015	Outpatient Research Identifiable File 2012-2015
Total ED visits	Count per person-year	HEDIS patient-level files 2012-2015	Outpatient Research Identifiable File 2012-2015
**3. Prescription drug use**			
Total Medicare Part D Drug Payments	$ per person-year	2012-2015 Master Beneficiary Summary File- Cost and Use supplement	2012-2015 Master Beneficiary Summary File- Cost and Use supplement
Total Beneficiary Drug Payments	$ per person-year	2012-2015 Master Beneficiary Summary File- Cost and Use supplement	2012-2015 Master Beneficiary Summary File- Cost and Use supplement
Part D Drug Fill Count (Derived measure indicating number of 30-day supply equivalents in each year)	Count per person-year	2012-2015 Master Beneficiary Summary File- Cost and Use supplement	2012-2015 Master Beneficiary Summary File- Cost and Use supplement
Total Medicare Part D Drug Cost	$ per person-year	2012-2015 Master Beneficiary Summary File- Cost and Use supplement	2012-2015 Master Beneficiary Summary File- Cost and Use supplement

Control variables

The main control variables will include age, gender, race and ethnicity, and other patient characteristics collected at the time of enrollment in the Look AHEAD trial, including clinical attributes (BMI, hemoglobin A1c, diabetes duration, and history of cardiovascular disease), education, and household income. We will also adjust for the study site.

d. Suggested data analysis plan

1. Compare matched and non-matched patients

First, we will compare the baseline characteristics of (1) Look AHEAD participants that consented to linkages and were matched to Medicare claims data versus (2) Look AHEAD participants that did not consent to linkages and (3) participants that consented but were not matched.

Next, we will investigate whether the observed baseline characteristics of matched ILI participants were similar to the observed baseline characteristics of matched DSE participants, in order to evaluate whether study attrition in our sample was non-random. We expect that non-random missingness is likely, given, for example, participants that died during the intervention period could not consent to linkage with Medicare databases.

We will repeat the main study analyses comparing weight, physical fitness, Hemoglobin A1c, and waist circumference between ILI and DSE participants during the intervention period for just the matched sample to determine whether the ILI had a similar impact relative to the overall sample.[2]

2. Descriptive analysis of timing of Medicare eligibility and per-enrollee Medicare spending

We have Medicare enrollment information for our sample from 2001 through 2015. We will determine the percentage of matched Look AHEAD participants enrolling in Medicare in each year and whether eligibility was due to age (i.e., age 65+), enrollment in Social Security Disability Insurance (reflecting that all Disability Insurance enrollees are eligible for Medicare after a 24-month qualifying period[5]), or End Stage Renal Disease (reflecting that individuals with ESRD are eligible for Medicare regardless of age[6]). We will calculate the percentage of ILI and control participants enrolling in traditional fee-for-service Medicare versus private Medicare Advantage plans. Finally, we will estimate total per-enrollee and total Medicare spending among fee-for-service enrollees in the ILI and control arms in each year between 2001 and 2015 using Cost and Utilization summary data.

3. Overall effects of ILI on Medicare outcomes

The main results will focus on the effects of ILI on Medicare outcomes between 2012-2015.

Reason for initial Medicare eligibility

We will investigate the effect of the ILI on whether initial eligibility for Medicare occurred through Disability Insurance and/or ESRD rather than through age-based eligibility. The unit of observation for this analysis will be a Look AHEAD participant. First, we will estimate the overall percentage of ILI and control participants initially eligible for Medicare through Disability Insurance or ESRD. Then, we estimate adjusted differences in eligibility categories controlling for baseline patient characteristics.

Hospital and emergency department use

We will investigate the effects of the ILI on the measures of hospital and emergency room use described above. The unit of observation for this analysis will be a participant-year, including data from 2012-2015. First, we will estimate rates of hospital and emergency department use separately for ILI and DSE participants over this period. Then we will estimate adjusted differences controlling for baseline patient characteristics.

Prescription drug use

We will estimate the effects of the ILI on measures of prescription drug use outlined above. The unit of analysis will be a participant-year, including data from 2012-2015. We will examine mean rates of prescription drug use and spending separately for ILI and control participants and then estimate adjusted differences controlling for baseline characteristics.

4. Year-specific effects of ILI on Medicare outcomes

Next, we will estimate year-specific ILI and control means and differences in overall hospital and ER use and prescription drug events between ILI and control participants for each year in our sample period (2012-2015). The year-specific differences will adjust for the same baseline characteristics as the overall estimates. These analyses will indicate whether ILI versus control differences stayed constant, widened, or narrowed between 2012 and 2015.

5. Overall effects stratified by baseline patient characteristics

We will investigate whether the effect of ILI on Medicare eligibility, hospital and emergency department use, and prescription drug use differs with baseline characteristics (i.e., at the time of study enrollment) that may be predictive of long-term health care use including socio-economic status (e.g., education and household income), BMI and other characteristics.

Finally, we will investigate whether the long-term effects of the ILI on hospitalizations, ED use, and prescription drug spending in 2012-2015 differ depending across ILI patients depending on the extent of weight loss and changes in diabetes control during the intervention period (i.e., between 2001 and prior to 2012).

e. Suggested table shells and figures

Figure 1. CONSORT Diagram

The consort diagram will include the following categories:

- Initial Look AHEAD Participants
- Look AHEAD Participants that died, dropped out, or lost to follow-up before Look AHEAD-C
- Look AHEAD Participants that reached Look AHEAD-C
- Look AHEAD Participants that consented to administrative linkages
- Look AEHAD Participants that did not consent to administrative linkages
- Look AHEAD Participants that consented to linkages and were successfully linked
- Look AHEAD Participants that consented to linkages and were not successfully linked

Table 1. Baseline Characteristics of Look AHEAD Participants Linked to Medicare Data

Characteristics	Controls (N=x)	ILI (N=x)	*P*-value
Age			
Sex			
Race/ethnicity			
Education			
Household income			
Physical fitness (METs)			
BMI			
Hemoglobin A1c			

Diabetes duration			
History of cardiovascular disease			
Health insurance status			
Usual source of health care			

Table 2: Health care utilization from 2012-2015

Characteristics	Controls (N=x)	ILI (N=x)	Adjusted Difference (SE)	*P*-value
**Original reason for Medicare eligibility**				
**Hospitalization outcomes**				
**Prescription drug use**				

Figure 2. Year-specific effects of the ILI on Medicare outcomes

There will be separate panels a-c for: hospital days/discharges, ER use, prescription drug events/costs

Each panel will display adjusted ILI versus control differences and confidence intervals for each year (2012-2015)

Table 3: Long-term Health Care Utilization by subgroup

(different panels for different subgroup analyses)

a. Baseline BMI (as an example)

Characteristics	Controls (N=x)	ILI (N=X)	Adjusted difference (SE)	P-value of ILI-control difference	*P*-value testing whether ILI-control differs across categories
**Reason for Medicare eligibility**					
25-29.9 kg/m^2					
30.0-39.9 kg/m^2					
>40.0 kg/m^2					
**Total hospitalizations**					
25-29.9 kg/m^2					
30.0-39.9 kg/m^2					

>40.0 kg/m^2					
**Total ED visits**					
25-29.9 kg/m^2					
30.0-39.9 kg/m^2					
>40.0 kg/m^2					
**Prescription drug spending**					
25-29.9 kg/m^2					
30.0-39.9 kg/m^2					
>40.0 kg/m^2					

Appendices

We anticipate that we will include a number of results as appendix tables or figures, as follows:

Tables

- Baseline characteristics of Look AHEAD participants that: (1) matched to Medicare data, (2) consented but did not match, and (3) did not consent for match.

Figures

- Percentage of matched Look AHEAD participants eligible for Medicare in each year overall and by initial eligibility category
- Percentage of ILI and control participants eligible for Medicare in each year overall and by initial eligibility category.
- Per-enrollee total Medicare spending for ILI and control participants in each year of the sample period.

f. Anticipated methodologic limitations or challenges, if present.

First, there may be differences in matched participants versus non-matched Look AHEAD participants. We can use inverse probability weights to balanced matched patients to approximate the distribution of the overall sample. Second, the observed covariates may differ between the ILI and control patient populations within the matched sample. We will adjust for baseline observed characteristics to address this issue.

21. **Will genetic data be used in this manuscript? (Please check one)**
_____ Yes __x__ No

22. **The lead author of this manuscript proposal has reviewed the list of existing Look AHEAD Study manuscript proposals and has found no overlap between this proposal and previously**

approved manuscript proposals either published or still in active status. For a list of proposals please go to: https://www.lookaheadtrial.org/secure/publications/pubListM.cfm

Please initial:
___PH___ Yes, I have reviewed the list of proposals and there is no apparent overlap.

23. **Is this manuscript proposal from a Look AHEAD ancillary study or use any ancillary study data? (Please check one.)**

__x__ Yes _____ No

24. **a. If yes, please list the ancillary study name and PI:**

The Long-Term Benefits of Interventions to Improve T2D Outcomes (PIs: Goldman and Huckfeldt)

b. If yes, please list the sites involved in the ancillary study along with the names of the site PIs:

___University of Minnesota (Peter Huckfeldt____

___University of Southern California (Dana Goldman)______________________________

___Wake Forest University (Nick Pajewski)____

____

____

____

____

____

PLEASE NOTE: Manuscript preparation is expected to be completed in one to three years. If a manuscript is not submitted for Look AHEAD P&P review within 2 years from the date of the approval, the manuscript proposal may be transferred to another author.

Please submit your proposal through the manuscript registry (www.lookaheadtrial.org). Dashboard>P&P>Add New Proposal. Contact Tara Beckner (tbeckner@wakehealth.edu) for assistance.

1. Look AHEAD Research Group. Look AHEAD (Action for Health in Diabetes): design and methods for a clinical trial of weight loss for the prevention of cardiovascular disease in type 2 diabetes. *Controlled Clinical Trials.* 2003;24:610-628.
2. Look AHEAD Research Group. Cardiovascular Effects of Intensive Lifestyle Intervention in Type 2 Diabetes. *New England Journal of Medicine.* 2013;369:145-154.
3. Look AHEAD Research Group. Long-term Effects of a Lifestyle Intervention on Weight and Cardiovascular Risk Factors in Individuals With Type 2 Diabetes Mellitus. *Archives of Internal Medicine.* 2010;170(17):1566-1575.
4. Espeland ME, Glick HA, Bertoni M, Brancati FL, Bray GA, et al. Impact of an Intensive Lifestyle Intervention on Use and Cost of Medical Services Among Overweight and Obese Adults With Type 2 Diabetes: The Action for Health in Diabetes. *Diabetes Care.* 2014;37:2548-2556.
5. Social Security Administration. Medicare for Working Beneficiaries with Disabilities. 2018; https://www.ssa.gov/disabilityresearch/wi/medicare.htm.
6. Medicare. Signing up for Medicare if you have ESRD. 2018; https://www.medicare.gov/information-for-my-situation/signing-up-for-medicare-if-you-have-esrd.
