# Supplementary material for: Associations of Intensive Lifestyle Intervention in Type 2 Diabetes With Health Care Use, Spending, and Disability: An Ancillary Study of the Look AHEAD Study
Source: JAMA Netw Open. 2020 Nov 24;3(11):e2025488. doi: 10.1001/jamanetworkopen.2020.25488 (PMC7686866; doi:10.1001/jamanetworkopen.2020.25488)
Supplement: Supplement 2. — eTable 1. Sample Sizes by Analytic Sample eTable 2. Baseline Characteristics of Full Sample and Linked Participants eTable 3. Health Care Use and Spending by Intervention Group, 2012 to 2015 eAppendix. Supplementary Methods and Results [file jamanetwopen-e2025488-s002.pdf]

Supplemental Online Content

Huckfeldt PJ, Frenier C, Pajewski NM, et al. Associations of intensive lifestyle intervention in type 2 diabetes with health care use, spending, and disability: an ancillary study of the Look AHEAD study. *JAMA Netw Open*. 2020;3(11):e2025488. doi:10.1001/jamanetworkopen.2020.25488

eTable 1. Sample Sizes by Analytic Sample

eTable 2. Baseline Characteristics of Full Sample and Linked Participants

eTable 3. Health Care Use and Spending by Intervention Group, 2012 to 2015

eAppendix. Supplementary Methods and Results

This supplemental material has been provided by the authors to give readers additional information about their work.

© 2020 Huckfeldt PJ et al. *JAMA Network Open.*

eTable 1. Sample Sizes by Analytic Sample

Sample	Control Person-Years	Intervention Person-Year	P value [a] (Intervention=Control)
**Overall person years in linked sample**			
**2012-2015**	**5,260**	**5,367**	
**2012-2014**	**3,883**	**3,960**	
**Enrolled in fee-for-service Medicare (Parts A and B) and Part D prescription drug coverage (Table 2)**			
Health care spending measures (2012-2015)	1,592 (30.3%)	1,662 (31.0%)	0.433
Health care use measures (2012-2014)	1,135 (29.2%)	1,192 (30.1%)	0.399
**Largest possible sample for each health care outcomes (eTable 3)**			
Hospital and emergency department use (2012-2014) (*Either: (1) enrolled in Medicare Parts A and B or (2) Medicare Advantage and present in HEDIS.)*	3,705 (95.4%)	3,792 (95.8%)	0.462
Fee-For-Service Spending *(Enrolled in fee-for service Medicare, Parts A and B)*	2,824 (53.6%)	2,874 (53.5%)	0.886
Prescription Drug Spending (*Enrolled in Medicare Part D*)	3,463 (65.8%)	3,594 (66.9%)	0.218

[a] P-value for test that probability of inclusion is the same between Intervention (ILI) and Control group.

© 2020 Huckfeldt PJ et al. *JAMA Network Open.*

eTable 2. Baseline Characteristics of Full Sample and Linked Participants

	**Full Look Ahead Sample**		**Linked Sample**	
	Control	Intervention	Control	Intervention
Number of participants	2,575	2,570	1,387	1,409
Age at baseline (years)	58.9	58.6	59.6	59.6
Female (%)	59.7	59.4	59.3	58.1
Race and ethnicity (%)				
White, non-Hispanic	63.3	63.1	66.0	66.6
Black, non-Hispanic	15.7	15.6	16.9	16.1
Hispanic	13.2	13.2	14.1	13.3
Asian/Pacific Islander	0.8	1.1	*	*
American Indian	5.0	5.1	*	*
Weight (lbs)	222.1	221.5	221.2	220.6
BMI	36	35.9	35.8	35.6
Obesity (%)				
Normal/Overweight	14.1	15.7	14.4	16.0
Obese	63.7	61.9	64.6	63.9
Very Obese	22.3	22.4	21.0	20.1
Hemoglobin A1c %	7.3	7.2	7.2	7.2
Elevated HbA1c (%)	55.2	53.4	52.8	51.0
Duration of Diabetes (years)	6.8	6.8	6.6	6.8
Hypertension (%)	82.8	83.6	84.3	83.3

© 2020 Huckfeldt PJ et al. *JAMA Network Open.*

History of CVD (%)	13.5	14.2	12.2	12.6
Employed (%)	62.6	63.3	66.0	63.2
Education (%)				
High school or less	22.5	21.6	21.9	21.1
Some post-secondary	37.6	36.8	36.8	34.0
College graduate or higher	39.9	41.5	41.3	44.9

Note: Asian or Pacific Islander and American Indian percentages were not included for the linked sample due to small cell sizes in order to comply with the CMS Cell Size Suppression Policy.

© 2020 Huckfeldt PJ et al. *JAMA Network Open.*

eTable 3. Health Care Use and Spending by Intervention Group, 2012 to 2015[a]

Outcome	Control Mean	Intervention Mean	Adjusted difference (95% CI)	Percentage difference (95% CI)[b]	P-value
**Hospital and emergency department use (2012-2014)**					
Any hospital admission (%)	13.4	13.2	-0.1 (-1.9, 1.6)	-0.8% (-13.9%, 12.3%)	0.910
Number of hospital admissions	0.19	0.19	0.01 (-0.03, 0.04)	3.4% (-15.8%, 22.6%)	0.729
Any ED visit (%)	19.5	19.6	0.2 (-1.9, 2.7)	0.8% (-9.7%, 11.3%)	0.883
Number of ED visits	0.28	0.28	-0.00 (-0.04, 0.04)	-0.3% (-15.0%, 14.3%)	0.967
**Fee-for-service Medicare spending (2012-2015)** [c,d]					
Part A FFS Spending ($)	5297	5453	67 (-913, 1046)	1.3% (-17.2%, 19.7%)	0.893
Part B FFS Spending ($)	3558	3911	293 (-63, 650)	8.2% (-1.8%, 18.3%)	0.107
**Prescription drug use (2012-2015)** [c]					
Total gross costs for Part D prescription drugs ($)	4708	4360	-538 (-993, -83)	-11.4% (-21.1%, -1.8%)	0.020
Total beneficiary payments for Part D prescription drugs ($)	1201	1072	-171 (-282, -60)	-14.2% (-23.5%, -5.0%)	0.002

[a] Outcomes include annual measures of health care use and spending. The estimation sample included 7497 person years for the hospital and emergency department analysis (this comprised 96% of linked person years from 2012-2014), 5698 person years for the fee-for-service Medicare spending analysis (available for 54% of linked person years), and 7057 person years for the Part D prescription drug analysis (available for 66% of linked person years).

[b] Reports adjusted difference and confidence interval divided by the control mean

[c] Spending results expressed in constant 2015 dollars.

[d] FFS stands for "fee-for-service".

© 2020 Huckfeldt PJ et al. *JAMA Network Open.*

eAppendix 1. Supplementary Methods and Results

1. Additional details of data linkage

Working with the CMS data contractor General Dynamics Information Technology (GDIT), we linked consenting Look AHEAD participants to Medicare databases using three identifiers: (1) Social Security Number, (2) Medicare number, and (3) last name, date of birth, and zip code. The third approach would only produce a linkage if there was a unique match.

For matches based on Social Security Number or Medicare number, we gauged whether a match was accurate based on whether there was correspondence between gender and date of birth between Look AHEAD data and CMS data. Among our final sample, 89% of matches agreed on both measures. In 11% of matches, we categorized a match as being successful if: 2 out of 3 birth date fields matched (i.e., month, day, and year) and gender matched OR if the birth date matched exactly but gender did not match. Notably, among participants with candidate matches using multiple identifiers, we always found agreement in the linkage (i.e., in terms of the linked identifier from GDIT).

We estimated the main analysis using just the 89% of perfect matches. We found a similar pattern of results, except that the main Part D spending results became statistically insignificant (with a similar estimated difference, but slightly wider confidence interval). All other significant results remained.

2. Coverage requirements for each outcome measure in Appendix Table A3.

Not all measures were available for all study participants, based on enrollment in traditional Medicare versus Medicare Advantage and enrollment in Part D drug coverage. We constructed measures of inpatient discharges and emergency department visits for 95.4% and 95.8% of linked person-years (in control and intervention groups, respectively). We were unable to construct these measures for some enrollees because we were not able to identify all Medicare Advantage enrollees in HEDIS, but there was no significant difference in our ability to construct outcome measures between the intervention and control groups (p=0.462, **Appendix Table A1**). We constructed measures of Part A and Part B spending for enrollees in fee-for-service Medicare with both Part A and Part B coverage, comprising 53.5 and 53.6% of person-years in the intervention and

© 2020 Huckfeldt PJ et al. *JAMA Network Open.*

control groups (p=0.886, **Appendix Table A1**). We were able to measure Part D gross drug costs for participants enrolled in Medicare Part D, comprising 65.8% and 66.9% of person-years (in control and intervention groups, respectively); this rate did not differ significantly between the control and intervention group (p=0.218, **Appendix Table A1**).

Many linked participants consented to linkages after the end of our study period and as a result only 1.4% of linked participants died during the sample period. We included person years when participants died in order to capture end-of-life spending in our analysis. We also included person years with less than 12 months of Medicare enrollment but controlled for enrolled months (Part A months for Part A spending, Part B months for Part B spending, and Part D months for Part D prescription drug costs and beneficiary out-of-pocket spending). For total spending, we controlled for Parts A, B, and D enrolled months. Finally, a small percentage person years (~6% in our primary analysis sample, equally distributed between intervention and controls) had both fee-for-service Medicare and Medicare Advantage enrollment in a given year. Our measures of annual Part A and B spending and total Medicare spending only captures the fee-for-service portion of spending. We kept these person years in the sample but also controlled for their months of Medicare Advantage (Part C) coverage. We continued to measure hospitalizations and ED visits in HEDIS (for Medicare Advantage months) and fee-for-service data (for fee-for service months).

3. Specification of general linear models

Estimating a general linear model requires specifying: (1) a "link" function (specifically the functional form for the outcome variable that best matches a linear combination of covariates) and (2) the appropriate distribution of the outcome variable. We used a "log" link function because the spending and visit outcome measures were highly skewed. We used modified Park tests to select the distributional family for each outcome, following the method described by Deb and Norton ("Modeling Health Care Expenditures and Use", Annual Review of Public Health, 39: 489-505; 2018). Based on the results, we specified a Poisson distribution for the number of hospital admissions and emergency department visits and a gamma distribution for each of the spending measures (Parts A, B, D and total Medicare spending).

© 2020 Huckfeldt PJ et al. *JAMA Network Open.*

4. Detailed estimates for effects of ILI on weight and HbA1c (displayed in Figure 2)

Linked participants had nearly identical weight during the baseline period (220.6 pounds for the ILI group versus 221.2 pounds for the control group). In the first year of the intervention, the control group lost 0.7% of body weight (95% CI: -1.0%, -0.5%; p<0.001) while the ILI group lost 9.0% of body weight (95% CI: -9.4%, -8.6%, p<0.001), resulting in a net weight loss of 8.3% (95% CI: -8.7, -7.8, p< 0.001) for the ILI group versus the control group. The ILI group also had a net reduction in HbA1c of 0.55 percentage points (95% CI: -0.62, -0.48, p<0.001) in the first year of the trial relative to the control group. Over the entire intervention period, the linked ILI group had a net weight loss of -3.7% (95% CI: -4.1, -3.2, p<0.001) and a net HbA1c reduction of 0.23 percentage points (95% CI: -0.30, -0.15, p<0.001) relative to the control group. Moreover, the ILI group maintained lower weight during the post-intervention period, as shown in Figure 2 for Years 11 and 12 after randomization. During our sample period from 2012-2015, the linked ILI group continued to have lower weight than the control group (adjusted difference: -1.9% of initial body weight, 95% CI: -2.6, -1.2, p<0.001). We found a similar effect for the primary analysis sample during this period (adjusted difference: -2.4% of initial body weight, 95% CI: -3.5, -1.2, p<0.001). However, there was no significant difference in HbA1c between 2012-2015 for either group.

5. Look AHEAD Research Group at End of Continuation

Clinical Sites

The Johns Hopkins University Frederick L. Brancati, MD, MHS[1*]; Jeanne M. Clark, MD, MPH[1] (Co-Principal Investigators); Lee Swartz[2]; Jeanne Charleston, RN[3]; Lawrence Cheskin, MD[3]; Richard Rubin, PhD[3*]; Jean Arceci, RN; David Bolen; Danielle Diggins; Mia Johnson; Joyce Lambert; Sarah Longenecker; Kathy Michalski, RD; Dawn Jiggetts; Chanchai Sapun; Maria Sowers; Kathy Tyler

*deceased

Pennington Biomedical Research Center George A. Bray, MD[1]; Allison Strate, RN[2]; Frank L. Greenway, MD[3]; Donna H. Ryan, MD[3]; Donald Williamson, PhD[3]; Timothy Church, MD[3]; Catherine Champagne, PhD, RD; Valerie Myers, PhD; Jennifer Arceneaux, RN; Kristi Rau; Michelle Begnaud, LDN, RD, CDE; Barbara Cerniauskas, LDN, RD, CDE; Crystal Duncan, LPN; Helen Guay, LDN, LPC, RD; Carolyn Johnson, LPN, Lisa

© 2020 Huckfeldt PJ et al. *JAMA Network Open.*

Jones; Kim Landry; Missy Lingle; Jennifer Perault; Cindy Puckett; Marisa Smith; Lauren Cox; Monica Lockett, LPN

The University of Alabama at Birmingham Cora E. Lewis, MD, MSPH[1]; Sheikilya Thomas, PhD,MPH[2]; Monika Safford, MD[3]; Stephen Glasser, MD[3]; Vicki DiLillo, PhD[3]; Gareth Dutton, PhD, Charlotte Bragg, MS, RD, LD; Amy Dobelstein; Sara Hannum; Anne Hubbell, MS; Jane King, MLT; DeLavallade Lee; Andre Morgan; L. Christie Oden; Janet Wallace, MS; Cathy Roche, PhD, RN, BSN; Jackie Roche; Janet Turman

Harvard Center

Massachusetts General Hospital. David M. Nathan, MD[1]; Enrico Cagliero, MD[3]; Heather Turgeon, RN, BS, CDE[2]; Barbara Steiner, EdM; Valerie Goldman, MS, RDN[2]; Linda Delahanty, MS, RDN[3]; Ellen Anderson, MS, RDN[3]; Laurie Bissett, MS, RDN; Christine Stevens, RN; Mary Larkin, RN; Kristen Dalton, BS, Roshni Singh, BS

Joslin Diabetes Center: Edward S. Horton, MD[1]; Sharon D. Jackson, MS, RD, CDE[2]; Osama Hamdy, MD, PhD[3]; A. Enrique Caballero, MD[3]; Sarah Bain, BS; Elizabeth McKinney, BSN, RN; Barbara Fargnoli, MS,RD; Jeanne Spellman, BS, RD; Kari Galuski, RN; Ann Goebel-Fabbri, PhD; Lori Lambert, MS, RD; Sarah Ledbury, MEd, RD; Maureen Malloy, BS; Kerry Ovalle, MS, RCEP, CDE

Beth Israel Deaconess Medical Center: George Blackburn, MD, PhD[1*] Christos Mantzoros, MD, DSc[3]; Ann McNamara, RN

*deceased

University of Colorado Anschutz Medical Campus James O. Hill, PhD[1]; Marsha Miller, MS RD[2]; Holly Wyatt, MD[3] , Brent Van Dorsten, PhD[3]; Judith Regensteiner, PhD[3]; Debbie Bochert; Gina Claxton-Malloy RD Ligia Coelho, BS; Paulette Cohrs, RN, BSN; Susan Green; April Hamilton, BS, CCRC; Jere Hamilton, BA; Eugene Leshchinskiy; Loretta Rome, TRS; Terra Thompson, BA, Kirstie Craul, RD, CDE; Cecilia Wang, MD

Baylor College of Medicine John P. Foreyt, PhD[1]; Rebecca S. Reeves, DrPH, RD[2]; Molly Gee, MEd, RD[2]; Henry Pownall, PhD[3]; Ashok Balasubramanyam, MBBS[3]; Chu-Huang Chen, MD, PhD[3]; Peter Jones, MD[3]; Michele Burrington, RD, RN; Allyson Clark Gardner, MS, RD; Sharon Griggs; Michelle Hamilton; Veronica Holley; Sarah Lee; Sarah Lane Liscum, RN, MPH; Susan Cantu-Lumbreras; Julieta Palencia, RN; Jennifer Schmidt; Jayne Thomas, RD; Carolyn White; Charlyne Wright, RN; Monica Alvarez, PCT

The University of Tennessee Health Science Center

University of Tennessee East. Karen C. Johnson, MD, MPH[□]; Karen L. Wilson, BSN[□]; Mace Coday, PhD[3]; Beate Griffin, RN, BS; Donna Valenski; Polly Edwards; Brenda Fonda; Kim Ward

University of Tennessee Downtown. Helmut Steinburg, MD[3]; Carolyn Gresham, BSN[□]; Moana Mosby, RN; Debra Clark, LPN; Donna Green RN; Abbas E. Kitabchi, PhD, MD (retired)

© 2020 Huckfeldt PJ et al. *JAMA Network Open.*

University of Minnesota Robert W. Jeffery, PhD[1]; Tricia Skarphol, MA[2]; John P. Bantle, MD[3]; J. Bruce Redmon, MD[3]; Richard S. Crow, MD[3]; Scott J. Crow, MD[3]; Manami Bhattacharya, BS; Cindy Bjerk, MS, RD; Kerrin Brelje, MPH, RD; Carolyne Campbell; Mary Ann Forseth, BA; Melanie Jaeb, MPH, RD; Philip Lacher, BBA; Patti Laqua, BS, RD; Birgitta I. Rice, MS, RPh, CHES; Ann D. Tucker, BA; Mary Susan Voeller, BA

St. Luke's Roosevelt Hospital Center Xavier Pi-Sunyer, MD[1]; Jennifer Patricio, MS[2]; Carmen Pal, MD[3]; Lynn Allen, MD; Janet Crane, MA, RD, CDN; Lolline Chong, BS, RD; Diane Hirsch, RNC, MS, CDE; Mary Anne Holowaty, MS, CN; Michelle Horowitz, MS, RD; Les James; Raashi Mamtani, MS

University of Pennsylvania Thomas A. Wadden, PhD[1]; Barbara J. Maschak-Carey, MSN, CDE[2]; Robert I. Berkowitz, MD[3]; Gary Foster, PhD[3]; Henry Glick, PhD[3]; Shiriki Kumanyika, PhD RD, MPH[3]; Yuliis Bell, BA ; Raymond Carvajal, PsyD; Helen Chomentowski; Renee Davenport; Lucy Faulconbridge, PhD;

Louise Hesson, MSN, CRNP; Sharon Leonard, RD; Monica Mullen, RD, MPH

University of Pittsburgh John M. Jakicic, PhD[1]; David E. Kelley, MD[1]; Jacqueline Wesche-Thobaben, RN, BSN, CDE[2]; Daniel Edmundowicz, MD[3]; Lin Ewing, PhD, RN[3]; Andrea Hergenroeder, PhD, PT, CCS[3]; Mary L. Klem, PhD, MLIS[3]; Mary Korytkowski, MD[3]; Andrea Kriska, PhD[3]; Lewis H. Kuller, MD, DrPH[3]; Amy D. Rickman, PhD, RD, LDN[3]; Rose Salata, MD[3]; Monica E. Yamamoto, DrPH, RD, FADA[3]; Janet Bonk, RN, MPH; Susan Copelli, BS, CTR; Rebecca Danchenko, BS; Tammy DeBruce, BA; Barbara Elnyczky; David O. Garcia, PhD; George A. Grove, MS; Patricia H. Harper, MS, RD, LDN; Susan Harrier, BS; Diane Heidingsfelder, MS, RD, CDE, LDN; Nicole L. Helbling, MS, RN; Diane Ives, MPH; Janet Krulia, RN, BSN, CDE; Juliet Mancino, MS, RD, CDE, LDN; Anne Mathews, PhD, RD, LDN; Lisa Martich, BS, RD, LDN; Meghan McGuire, MS; Tracey Y. Murray, BS; Anna Peluso, MS; Karen Quirin; Jennifer Rush, MPH; Joan R. Ritchea; Linda Semler, MS, RD, LDN; Karen Vujevich, RN-BC, MSN, CRNP; Kathy Williams, RN, MHA; Donna L. Wolf, PhD

The Miriam Hospital/Brown Medical School Rena R. Wing, PhD[1]; Renee Bright, MS[2]; Vincent Pera, MD[3]; Deborah Tate, PhD[3]; Amy Gorin, PhD[3]; Kara Gallagher, PhD[3]; Amy Bach, PhD; Barbara Bancroft, RN, MS; Anna Bertorelli, MBA, RD; Richard Carey, BS; Tatum Charron, BS; Heather Chenot, MS; Kimberley Chula-Maguire, MS; Pamela Coward, MS, RD; Lisa Cronkite, BS; Julie Currin, MD; Maureen Daly, RN; Caitlin Egan, MS; Erica Ferguson, BS, RD; Linda Foss, MPH; Jennifer Gauvin, BS; Don Kieffer, PhD; Lauren Lessard, BS; Deborah Maier, MS; JP Massaro, BS; Tammy Monk, MS; Rob Nicholson, PhD; Erin Patterson, BS; Suzanne Phelan, PhD; Hollie Raynor, PhD, RD; Douglas Raynor, PhD; Natalie Robinson, MS, RD; Deborah Robles; Jane Tavares, BS

The University of Texas Health Science Center at San Antonio Helen P. Hazuda, PhD[1]; Maria G. Montez, RN, MSHP, CDE[2]; Carlos Lorenzo, MD[3]; Charles F. Coleman, MS, RD; Domingo Granado, RN; Kathy Hathaway, MS, RD; Juan Carlos Isaac, RC, BSN; Nora Ramirez, RN, BSN

© 2020 Huckfeldt PJ et al. *JAMA Network Open.*

VA Puget Sound Health Care System / University of Washington Steven E. Kahn, MB, ChB[1]; Anne Kure, BS[2]; Edward J. Boyko, MD, MPH[3]; Edward Lipkin, MD, PhD[3]; Dace Trence, MD[3]; Subbulaxmi Trikudanathan, MD, MRCP, MMSc[3]; Elaine Tsai, MD[3]; Brenda Montgomery, RN, MS, CDE; Ivy Morgan-Taggart; Jolanta Socha, BS; Lonnese Taylor, RN, BS; Alan Wesley, BA

Southwestern American Indian Center, Phoenix, Arizona and Shiprock, New Mexico William C. Knowler, MD, DrPH[1]; Paula Bolin, RN, MC[2]; Tina Killean, BS[2]; Maria Cassidy-Begay, BSND, RND [2]; Katie Toledo, MS, LPC[2]; Cathy Manus, LPN[3]; Jonathan Krakoff, MD[3]; Jeffrey M. Curtis, MD, MPH[3]; Sara Michaels, MD[3]; Paul Bloomquist, MD[3]; Peter H. Bennett, MB, FRCP[3]; Bernadita Fallis, RN, RHIT, CCS; Diane F. Hollowbreast; Ruby Johnson; Maria Meacham, BSN, RN, CDE; Christina Morris, BA; Julie Nelson, RD; Carol Percy, RN, MS; Patricia Poorthunder; Sandra Sangster; Leigh A. Shovestull, RD, CDE; Miranda Smart; Janelia Smiley; Teddy Thomas, BS

University of Southern California Anne Peters, MD[1]; Siran Ghazarian, MD[2]; Elizabeth Beale, MD[3]; Kati Konersman, RD, CDE; Brenda Quintero-Varela; Edgar Ramirez; Gabriela Rios, RD; Gabriela Rodriguez, MA; Valerie Ruelas MSW, LCSW; Sara Serafin-Dokhan; Martha Walker, RD

Coordinating Center

Wake Forest University Mark A. Espeland, PhD[1]; Judy L. Bahnson, BA, CCRP[3]; Lynne E. Wagenknecht, DrPH[1]; David Reboussin, PhD[3]; W. Jack Rejeski, PhD[3]; Alain G. Bertoni, MD, MPH[3]; Wei Lang, PhD[3]; David Lefkowitz, MD[3*] Patrick S. Reynolds, MD[3]; Denise Houston, PhD[3]; Mike E. Miller, PhD[3]; Laura D. Baker, PhD[3]; Nicholas Pajewski, PhD[3]; Stephen R. Rapp, PhD[3]; Stephen Kritchevsky, PhD[3]; Haiying Chen, PhD, MM[3]; Valerie Wilson, MD[3]; Delia S. West, PhD[3]; Ron Prineas, MD[3]; Tandaw Samdarshi, MD[3]; Amelia Hodges, BS, CCRP[2]; Karen Wall[2]; Carrie C. Williams, MA, CCRP[2]; Andrea Anderson, MS; Jerry M. Barnes, MA; Tara D. Beckner; Delilah R. Cook; Valery S. Effoe, MD, MS; Melanie Franks, BBA; Katie Garcia, MS; Sarah A. Gaussoin, MS; Candace Goode; Michelle Gordon, MS; Lea Harvin, BS; Mary A. Hontz, BA; Don G. Hire, BS; Patricia Hogan, MS; Mark King, BS; Kathy Lane, BS; Rebecca H. Neiberg, MS; Julia T. Rushing, MS; Debbie Steinberg, BS; Jennifer Walker, MS; Michael P. Walkup, MS

*deceased

Central Resources Centers

Central Laboratory, Northwest Lipid Metabolism and Diabetes Research Laboratories Santica M. Marcovina, PhD, ScD[1]; Jessica Hurting[2]; John J. Albers, PhD[3], Vinod Gaur, PhD[4]

ECG Reading Center, EPICARE, Wake Forest University School of Medicine

Elsayed Z. Soliman MD, MSc, MS[1]; Charles Campbell [2]; Zhu-Ming Zhang, MD[3]; Mary Barr; Susan Hensley; Julie Hu; Lisa Keasler; Yabing Li, MD

Hall-Foushee Communications, Inc.

Richard Foushee, PhD; Nancy J. Hall, MA

© 2020 Huckfeldt PJ et al. *JAMA Network Open.*

Federal Sponsors

National Institute of Diabetes and Digestive and Kidney Diseases Mary Evans, PhD; Van S. Hubbard, MD, PhD; Susan Z. Yanovski, MD

National Heart, Lung, and Blood Institute Lawton S. Cooper, MD, MPH; Peter Kaufman, PhD, FABMR; Mario Stylianou, PhD

Centers for Disease Control and Prevention Edward W. Gregg, PhD; Ping Zhang, PhD

Funding and Support

Funded by the National Institutes of Health through cooperative agreements with the National Institute of Diabetes and Digestive and Kidney Diseases: DK57136, DK57149, DK56990, DK57177, DK57171, DK57151, DK57182, DK57131, DK57002, DK57078, DK57154, DK57178, DK57219, DK57008, DK57135, and DK56992. Additional funding was provided by the National Heart, Lung, and Blood Institute; National Institute of Nursing Research; National Center on Minority Health and Health Disparities; NIH Office of Research on Women's Health; and the Centers for Disease Control and Prevention. This research was supported in part by the Intramural Research Program of the National Institute of Diabetes and Digestive and Kidney Diseases. The Indian Health Service (I.H.S.) provided personnel, medical oversight, and use of facilities. The opinions expressed in this paper are those of the authors and do not necessarily reflect the views of the I.H.S. or other funding sources.

Additional support was received from The Johns Hopkins Medical Institutions Bayview General Clinical Research Center (M01RR02719); the Massachusetts General Hospital Mallinckrodt General Clinical Research Center and the Massachusetts Institute of Technology General Clinical Research Center (M01RR01066); the Harvard Clinical and Translational Science Center (RR025758-04); the University of Colorado Health Sciences Center General Clinical Research Center (M01RR00051) and Clinical Nutrition Research Unit (P30 DK48520); the University of Tennessee at Memphis General Clinical Research Center (M01RR0021140); the University of Pittsburgh General Clinical Research Center (GCRC) (M01RR000056), the Clinical Translational Research Center (CTRC) funded by the Clinical & Translational Science Award (UL1 RR 024153) and NIH grant (DK 046204); the VA Puget Sound Health Care System Medical Research Service, Department of Veterans Affairs; and the Frederic C. Bartter General Clinical Research Center (M01RR01346).

The following organizations have committed to make major contributions to Look AHEAD: FedEx Corporation; Health Management Resources; LifeScan, Inc., a Johnson & Johnson Company; OPTIFAST® of Nestle HealthCare Nutrition, Inc.; Hoffmann-La Roche Inc.; Abbott Nutrition; and Slim-Fast Brand of Unilever North America.

Some of the information contained herein was derived from data provided by the Bureau of Vital Statistics, New York City Department of Health and Mental Hygiene.

© 2020 Huckfeldt PJ et al. *JAMA Network Open.*

[1] Principal Investigator

[2] Program Coordinator

[3] Co-Investigator

All other Look AHEAD staffs are listed alphabetically by site.

© 2020 Huckfeldt PJ et al. *JAMA Network Open.*
