# Supplementary material for: Associations of Intensive Lifestyle Intervention in Type 2 Diabetes With Health Care Use, Spending, and Disability: An Ancillary Study of the Look AHEAD Study
Source: JAMA Netw Open. 2020 Nov 24;3(11):e2025488. doi: 10.1001/jamanetworkopen.2020.25488 (PMC7686866; doi:10.1001/jamanetworkopen.2020.25488)
Supplement: Supplement 3. — Data Sharing Statement [file jamanetwopen-e2025488-s003.pdf]

Data Sharing Statement

Huckfeldt. Associations of Intensive Lifestyle Intervention in Type 2 Diabetes With Health Care Use, Spending, and Disability. *JAMA Netw Open*. Published November 24, 2020. doi:10.1001/jamanetworkopen.2020.25488

Data
Data available: No

Additional Information
Explanation for why data not available: We accessed our data through a data use agreement with CMS, which does not allow us to share our data.
